# Dimensions of social and political capital in interventions to improve household well-being: Implications for coffee-growing areas in southern Colombia

**DOI:** 10.1371/journal.pone.0245971

**Published:** 2021-01-25

**Authors:** Adriana E. Suárez, Isabel Gutiérrez-Montes, Fausto Andres Ortiz-Morea, Claudia Ordoñez, Juan Carlos Suárez, Fernando Casanoves

**Affiliations:** 1 Facultad de Ciencias Agropecuarias, Programa de Doctorado Ciencias Naturales y Desarrollo Sustentable, Universidad de la Amazonia, Florencia (Caquetá), Colombia; 2 Facultad de Ciencias Agropecuarias, Programa de Maestría en Sistema Sostenibles de Producción, Universidad de la Amazonia, Florencia, Colombia; 3 Centro de Investigaciones Amazónicas CIMAZ Macagual Cesar Augusto Estrada González, Grupo de Investigaciones Agroecosistemas y Conservación en Bosques Amazónicos- GAIA, Universidad de la Amazonia, Florencia, Colombia; 4 CATIE-Centro Agronómico Tropical de Investigación y Enseñanza, Turrialba, Costa Rica; 5 Facultad de Ingeniería, Programa de Ingeniería Agroecológica, Universidad de la Amazonia, Florencia, Colombia; 6 Centro de Formación Agroindustrial “La Angostura” SENA, Campoalegre, Huila, Colombia; Wroclaw University of Economics and Business, POLAND

## Abstract

This paper studies the influence of community capitals on well-being through a Community Capital Index (CCI) within coffee-growing families in southern Colombia. Our results show different farm typologies, with different levels of capital endowment translated into well-being that, in our case, were represented in the CCI. Specifically, social and political capitals positively affect coffee-growing families’ decisions in terms of life strategies. The results of this study increase our understanding of welfare enhancement and its relationship with capital endowment according to the type of coffee producer, having implications for the planning of more effective programs towards the improvement of quality of life.

## Introduction

Coffee cultivation is the second most commercialized product in the world after oil and is produced in countries of the tropics [1]. In Colombia, this crop is considered one of the main lines of the economy [2,3], representing 22% of the agricultural GDP (Gross domestic product) with a production estimated of 876 million kg year^-1^, which represents the sustaining of 530,000 rural families (25% of the rural population), distributed in 20 departments, with 588 coffee growing municipalities [4,5].

The coffee activity in different countries, including Colombia, promotes a peasant and family economy [6], generating about 0.8 and 1.4 million direct and indirect jobs, respectively. In this sense, agricultural activities offer about 32% of employment [7], being coffee cultivation the means of subsistence that affects the living conditions of the different typologies of coffee producers [8–10]. Living conditions related to the quality of life and well-being at the family and farm level are influenced by different characteristics such as education level. Education has been a variable of human capital that has a high correlation with the adoption of technology, higher productivity, and economic growth [6].

In the department of Huila (Colombia), coffee-growing activity has considerable social and economic importance due to the number of families that depend on it. It is developed in 101,630 farms by 82,763 coffee growers (14% of the total number of people that carry out the coffee activity in Colombia) in 152,427 ha planted (15% of the total planted area in Colombia) [7]. Besides, production in the department of Huila is classified as specialty coffee, allowing for the generation of a differentiated product with added value [11], increasing the competitiveness of the producers, which has impacted the improvement of the quality of life of coffee-growing families.

One method proposed to carry out processes of characterization of families based on the generation of typologies is using the Community Capitals Framework (CCF) [12], and on the work of Scoones [13], who was the first to provide an analytical framework for sustainable rural livelihoods. The livelihoods comprise the assets of natural, physical, human, financial, and social capitals; meanwhile, the activities and the access to assets jointly determine the living gained by the individual or household [14–16]. The CCF is based on the characterization of assets and capital endowments and the description of households’ living strategies, making it possible to identify the different components and technological innovations used in production systems [6,17,18].

Well-being refers to the positive and desirable condition of life [19]. The well-being considers various elements beyond economic (income and costs) and relates to the level of food security, education, security, environmental integrity, social relations, and freedom [20]. Some studies have measured well-being using different elements such as housing conditions, health care, education and knowledge, psychological conditions, and social interaction [21]. Some authors consider only health and housing for measuring well-being, and that the application of a large number of elements could turn difficult the measuring of well-being [22]. The well-being of rural households is linked to a number of specific community indicators, which are related to inequalities in household asset endowment. This inequality in capital endowment is influenced by the variation in sub-welfare at the level of families in the same area [23]. Therefore, the differences between rural households and communities that have generated livelihood diversification continually require innovative analytical approaches to determine well-being outcomes [24]. Within these analytical approaches, the CCF considers capitals stocks and flows of value, which may be converted into other forms of stocks and flows of value [12,17,24]. CCF is an approach that allows measuring the well-being of families, going further than the economic approach since it measures other elements such as the natural, political and social components [23].

Currently, there is a need to value and characterize the most important production systems, such as coffee, from the identification of community capitals, which is based on the recognition of interdependence, interaction, and synergy between capitals [24]. This incidence is called the "spiral of capitals" since there is a relationship with the development of communities, and this spiral can be oriented in an ascending or descending way [25], considering that the use of the assets in a single capital can have a positive or negative effect on other capitals. Therefore, the loss or degradation of assets within a capital would negatively affect one or more capitals, severely depleting, and compromising the community’s health and sustainability [24,26]. This study aims to characterize the different typologies of coffee producers in southern Colombia as the variables that positively or negatively impact the level of well-being using the spiral of capital approach. The CCF was used to understand the flow between capitals as a result of the interaction between coffee activity and livelihoods and to analyze how the impacts of this flow affect the system in terms of the particular characteristics of each of the typologies of coffee producers [12,17,24]. In this sense, we set out to answer the following questions: i. which kinds of Community Capitals are most important in differentiating among types of coffee farmers? ii. which kinds of Community Capitals are most important in determining well-being, and how can well-being be measured? iii. how do differential endowments of Capitals lead to upward or downward spiral?, and iv. what is the specific impact of key elements of coffee cultivation on well-being and environmental sustainability? The information generated in our research will allow us to focus our efforts on families that need to increase the endowment of some capital, specifically the critical aspects of coffee cultivation, for example, producer associations, community boards, and certified/organic production that impact the welfare of families and environmental sustainability.

## Materials and methods

### Area of study and data collection

The study was conducted in the municipality of Pitalito, located in the southeast of the department of Huila. This municipality has an extension of 625.5 km^2^ (1° 51’ 07" North Latitude and 76° 02’ 14" West Longitude), with a population of 125,000 habitants, a rural zone conformed by eight townships and 136 villages, and is considered the main coffee producing municipality in Colombia. Pitalito is located in the Colombian massif, close to the snowy mountains that occasionally emits ashes that subsequently are fixed in the soil, influencing the characteristics of flavor and aroma to the coffee. In this municipality, besides being cultivated the variety Colombia, which was the most known among the robust coffees, other types of coffee as the supreme one, and the gueicha coffee, an exotic coffee with flavors of chili, pepper, lemongrass and aromatic plants are also cultivated.

Households (n = 97) were randomly selected from the list of existing coffee producer members in the municipality of Pitalito, who were contacted through the president of the association. After accepting to participate in the study, each of the coffee family’s farms was visited for the survey, which lasted an average of 90 minutes and was made up of different sections that included (1) type of land tenure; (2) family characteristics; (3) land use; (4) management of coffee cultivation; (5) production of coffee beans; (6) PES (payment for environmental services); (7) sale of coffee; (8) cost of production; (9) equipment and infrastructure; (10) labor; (11) associativity. The survey questions were related to each of the community capitals with multiple choice responses. It was verified that coffee production is one of the principal livelihoods for the families surveyed who have developed this activity in the last 30 years, being this a principal economic driver. They are families whose origin is from other municipalities of the department of Huila, as well as from other departments of the south of Colombia with coffee culture such as Cauca, Nariño, Antioquia, among others, that due to the coffee bonanza of the ’80s, decided to invest in the planting of this crop.

### Methods

Seven types of capitals were analyzed: Natural, Human, Social, Cultural, Political, Physical, and Financial [12,17,25,27] using mixed research methods (quantitative and qualitative methodologies). The capitals allowed to characterize the available assets of the different types of coffee-producing families (Table 1).

**Table 1:** A general classification of the capital or resources involved in the generation of typologies.

FACTORS	CAPITAL	DEFINITION	EXAMPLE
**Humans**	Cultural	Reflects how community members view the world and how they act upon it	Includes cultural management of the crop, customs, and definition of what can and should be changed
	Human	Characteristics of people that facilitate their ability to develop a certain life strategy	Education, Participation, Workforce, Family Composition
	Social	Resources that include building support networks, membership in organized groups, and relationships of trust	Partnership, organizational benefits, sense of belonging and identity
	Political	The ability of an individual or group to influence resource mobilization or decision making	Participation in decision making, relationship with authorities, organization of the bases
**Materials**	Physical-Constructed	Basic infrastructure to support the production of goods or to improve the quality of life of people	Housing, basic services, technological level, structural level, distance to town
	Natural	Includes all-natural resources that generate goods and services or add up to more resources to support a livelihood	Forest, crops, ecosystem services, processes that generate pollution
	Financial	Any financial resource that people use to develop a livelihood.	Productive activities, costs, expenses, savings, credits

Sources: Adapted from [13,14,15,19].

### Statistical analysis

The classification of the types of coffee families was based on a matrix of 66 variables (number of variables for each capital: Human 15, Cultural 13, Social 4, Political 3, Physical 6, Natural 5 and Financial 20) characterized for each family (Table 2). Hierarchical cluster analysis (Ward method and Euclidean distance) was used to define coffee producers’ typologies. In order to determine the significance of the groups formed, a multivariate analysis of variance was performed, and later a gDGC mean vectors comparison [28] with a significance level of 0.05 was used to compared the groups mean vector. To identify which original variables best characterize coffee families’ typologies found in the cluster analysis, ANOVA was performed for each of the 66 variables using as classification criteria the typologies defined. For means comparison after reject the ANOVA null hypothesis a Fisher LSD test was used (p<0.05) General and mixed linear models (GLM) were used to determine differences between typologies for 38 variables, and Generalized Linear Mixed Models (GLMM) were used for 24 count variables. The association between the 4 presence-absence variables and typologies were analyzed using contingency tables. Additionally, from the Principal Component Analysis (PCA), the variables with the greatest contribution in components 1 and 2 were identified. To determine the synergies and trade-offs between capitals of the communities, with the variables that presented significant differences between coffee producers’ typologies, a Multiple Factor Analysis (MFA) was performed where those with the highest contribution by each capital were identified. Likewise, a co-inertia analysis was carried out to determine the relationship between the matrixes of variables for each capital. Subsequently, each capital variable that presented significant differences between the typologies was transformed into values in the interval of 0–1, where zero corresponds to the lowest value and one to the highest value observed. For each capital, a sub-index was constructed from the unweighted sum of its transformed variables (0–1). Adding the value of each sub-index per capital and mapping it to an interval of 0–1, the Community Capital Index (CCI) was constructed, which shows the degree of endowment and well-being of coffee-growing families. For each capital sub-indicator and the CCI, an analysis of variance was performed using the Fisher LSD test p<0.05 to determine differences between typologies. Based on what was proposed by Gutierrez et al. [27] and Emery and Flora [25] for the selection of the variables that have an impact on the "spiral of capitals", those that had both a positive and negative impact on the variation of the Community Capital Index (CCI) were considered. These variables are those that allow a change of the coffee families in the typologies. The principal components analysis, cluster, analysis of variance and regression, general and mixed linear models, generalized linear mixed models, and contingency tables were carried out using the InfoStat program [29]. The association between the Community Capital Index (CCI) and each capital was analyzed by calculating Pearson correlation coefficients. To visualize the correlations, chord diagrams were elaborated using the *circlize* packages [30]. The *FactoMineR* package [31] was used to obtain the hierarchical clustering graphs on the factor map and multiple factor analysis. The ACP plots were made using the *Ade4* package [32] of R version 3.4.3 [33].

**Table 2:** Matrix of 66 variables used to define (number of variables for each capital: Human 15, Cultural 13, Social 4, Political 3, Physical 6, Natural 5 and Financial 20).

Capital	variable	abbreviation	Unity	Description
**HUMAN**	Age of head of household	AHH	Number of years	The time that has lived the head of the household from his/her birth.
	Years of education of the head of household	YEH	Number of years	It was determined according to the average of studied years of head of the household. Six levels of education levels were established: a) uncompleted primary education (2,5 years); b) completed primary education (5 years); c) uncompleted secondary education (7 years); d) completed secondary education (11 years); and) technical education (12 years); and f) undergraduate degree (16 years).
	Training Assistance	TA	Number of training courses	It was obtained from the information supplied by the families that go for training courses. The considered organizations were: 1. the federation of coffee growers, 2. companies, and 3. associations. The recognized subjects were: 1. agricultural practices, 2. crops management, 3. environment, 4. water.
	Total labor force	ToL	Number of daily wages	It was obtained by adding the total of work that the producers do for the crop management of coffee. It is expressed in daily wages.
	Labor fertilization/year	LFe	Number of daily wages	It was determined from the producer’s information related to the daily wages needed for fertilization activities.
	Labor application herbicide/year	LHe	Number of daily wages	It was determined from the information supplied by the producer related to the daily wages needed for herbicide application.
	Labor disease management	LDM	Number of daily wages	It was determined from the information supplied by the producer related to the daily wages needed for disease management
	Labor Pest Management	LPM	Number of daily wages	It was determined from the information supplied by the producer related to the daily wages needed for pest management
	Labor collection	LCo	Number of daily wages	It was determined from the information supplied by the producer related to the daily wages needed for harvesting
	Labor Benefit	LBe	Number of daily wages	It was determined from the information supplied by the producer related to the daily wages needed for benefit activities
	Family Size	FSi	Number of persons	Total members of the family.
	Number of men	NuM	Number of men	quantity of males that are part of the family
	Number of women at home	NuW	Number of women	quantity of females that are part of the family
	Number of people who can read	NPR	Number of persons	Quantity of family members that can read
	Family’s average level of education	FLE	Number of years	It was determined according to the average of studied years of family members able to go to an educational center (< 12 years). Six levels of education levels were established: a) uncompleted primary education (2,5 years); b) completed primary education (5 years); c) uncompleted secondary education (7 years); d) completed secondary education (11 years), e) technical education (12 years); and f) undergraduate degree (16 years).
**CULTURAL**	Coverage Type Management	CTM	Number of coverages	It was calculated considering the number of coverage used by the producers such as 1) azuelda, 2) leaf litter, 3) Arachis pintoi, and 4) others.
	Soil conservation practice management	SCP	Number of practices	It was calculated considering the number of soil conservation practices implemented by the producers such as 1) ditches, 2) living barriers, 3) diversion channels, 4) contour lines, 5) sowing across the slope.
	Agroforestry practice management	APM	Number of practices	It was calculated considering the number of agroforestry practices implemented by the producers such as 1) live fences, 2) windbreaks, 3) scattered trees, 4) trees for crop surplus, 5) protein bank, 6) home gardens, and 7) fallows.
	Frequency per year of weed control chemically	YCC	Number of practices	It corresponds to the times per year that weed control chemically is done.
	Frequency per year of fertilization of coffee plantations	FYF	Number of doses	It corresponds to the total doses applied by the producers
	Availability of soil analysis	Asa	Number of analysis	It was scored 1 if the producer has a soil analysis and 0 if not.
	Frequency per year of foliar fertilization	YFF	Number of doses	It corresponds to the times per year that foliar fertilization is done.
	Type of organic fertilizers management	OFM	Number of types	It was calculated considering the number of organic fertilizers used by the producers such as 1) chicken manure, 2) compost, 3) vermicompost and 4) others
	Organic fertilization dose per year	OFY	Number of doses	It corresponds to the total doses of organic fertilizers applied by the producers
	Diseases management	DMa	Number of diseases	It was scored 1 if the producer does disease management and 0 if not.
	Pest Management	PMa	Number of pests	It was calculated considering the number of practices that the producer does to battle pests as 1.broca 2. coffee leaf miner 3. cochineal or moth 4. nematodes and 5. spider mite
	Frequency per year of pruning to the shade	YPS	Number of pruning	It corresponds to the times per year that pruning is done.
	Pruning management to coffee plantation	MCP	Number of pruning	It was scored 1 if the producer does pruning management and 0 if not.
**SOCIAL**	Technical assistance	Tas	Number of attendances	It was calculated considering the number of organizations that offer this service to the producers,such as 1. the federation of coffee growers, 2. companies, and 3. associations, and 4. others.
	Benefits of the National Federation of Coffee Growers	BFNc	Number of benefits	It was calculated considering the number of benefits offered by the FNC for the producers such as 1. economics, 2. technics, 3. FInancial.
	Associativity	Aso	Number of associations	It was calculated considering the number of organizations to which the producer belongs, such as Agroempresarial, Apecafe, Aprofusa, Ashulcafe, Saint Association Roque, Cadefihuila, Asoprocapi, Andean Group, Saint Mateo, Saint Isidro, Palmar Of the Creole, Fenace, and Fedecafe.
	Membership time in years to associations	MYA	Number of years	Time in years of the membership of the producer.
**POLITICAL**	Belonging to a community action board	CAB	Number of affiliations	It was scored 1 if the producer belongs to a community action board and 0 if not.
	Participation in activities of the community action board of the village	PAC	Number of units	It was scored 1 if the producer participates in the community action board’s activities and 0 if not.
	Coffe producer ID	CPId	Number of IDs	It was scored 1 if the producer has coffee producer ID and 0 if not.
**PHYSICAL**	Property Size	PSz	Ha	Extension in hectares of the property
	Distance nearest town	DNT	Km	Distance in Kilometers from the nearest town
	Level of basic services	LBS	Number of services	It was calculated according to the availability of the basic services in the property such as 1. water, 2. electricity, 3. telephone, 4. cellular and 5. internet.
	The technological level of the farm	TLF	Number of technologies	It was determined according to the availability of machinery and equipment for agricultural activities such as 1. soil drill, 2. pump of Back, 3. pump with an engine, 4. stationary pump, 5. demucilagator, 6. scythe, 7. pulper, 8. chainsaw, and 9. others.
	Level of tools on the farm	LTF	Number of tolos	It was determined according to the availability of tools for agricultural activities such as 1. wide shovels, 2. spadesful, 3. machete, 4. handsaw, 5. wheelbarrow, 6. abrasive file, 7. collector, 8. security equipment, 9. bars, and 10. hoes.
	Structural technological level	STL	Number of technologies	It was determined according to the availability of infrastructure on each farm, such as 1. the beneficiary, 2. cellar of coffee, 3. cellar of supplies, 4. camp or accommodations, 5. houses, 6. other constructions, and 7. dryer.
**NATURAL**	Process that generates pollution	PGP	Number of processes	It was determined according to activities that generate pollution such as 1. inappropriate management of rubbishes 2. absence of septic tank 3. inappropriate management of coffee honey, an 4. inappropriate management of agrochemical waste.
	Ecosystem services provided by your farm	ESf	Number of services	It was determined according to the ecosystem services provided by the farm such as 1. conservation of the biodiversity, 2. landscaping, 3. CO2 capture, 4. ecotourism, 5. food, and 6. water supply.
	Land use for crops	LUC	Ha	Area of the farm used for semiannual or perennial crops.
	Forest land use	FLA	Ha	Area of the farm kept as forest
	Land use for coffee production	LUCp	Ha	Area of the farm used for coffee production.
**FINANCIAL**	Hectare value	HeV	thousands of dollars	Value of a hectare of the farm according to the producer
	Cost Property size	CPS	thousands of dollars	Value of the entire property according to the producer
	Coffee production	CPD	Ha	Value of the coffee production according to the area that is in production
	Access to credits	ACC	No. of credits	Times that the producer has acquired a loan
	Certification of the coffee plantation	CCP	No. of certifications	It was scored 1 if the coffee plantation has a certification and 0 if not.
	Labor cost of fertilization application	LCF	thousands of dollars	It was calculated by multiplying the amount of labor force needed for fertilization by the price of daily wage.
	Cost of labor handling herbicides	LHH	thousands of dollars	It was calculated by multiplying the amount of labor force needed for herbicide application by the price of daily wage.
	Labor cost diseases management	CDM	thousands of dollars	It was calculated by multiplying the amount of labor force needed for disease management by the price of daily wage.
	Cost of labor pest management	CPM	thousands of dollars	It was calculated by multiplying the amount of labor force needed for pest management by the price of daily wage.
	Harvest labor cost	HLC	thousands of dollars	It was calculated by multiplying the amount of labor force needed for harvesting by the price of daily wage.
	Benefit labor cost	BLC	thousands of dollars	It was calculated by multiplying the amount of labor force needed for benefit activities by the price of daily wage.
	Income for wet coffee/farm	IWC	thousands of dollars	It was calculated by multiplying the total production of wet coffee by the load price according to the values reported by the producers.
	Income for dry parchment coffee/farm	IDP	thousands of dollars	It was calculated by multiplying the total production of dry parchment coffee by the load price according to the values reported by the producers.
	Cost of fertilizers	CFe	thousands of dollars	Amount of money spent on fertilizers according to the producer
	Income from other agricultural activities	IOAa	thousands of dollars	It was estimated according to the producers’ incomes related to agricultural activities other than coffee production.
	Income for special dry parchment coffee/farm	ISDc	thousands of dollars	It was determined from the values reported by the families regarding the sale of coffee special dry parchment
	Income for pasilla/farm	IPF	thousands of dollars	It was determined from the values reported by the families regarding the sale and self-consume of coffee products.
	Total income for coffee	TIC	thousands of dollars	It was calculated by adding the amount of money received by sales of 1) wet coffee, 2. dry parchment coffee, 3. pasilla and 4. special dry parchment.
	Cost of herbicides	CoH	thousands of dollars	Amount of money spent on herbicides according to the producer
	Costs of fungicide	CoF	thousands of dollars	Amount of money spent on fungicides according to the producer

## Results

### Typology of coffee producers in southern Colombia

**Fig 1:**
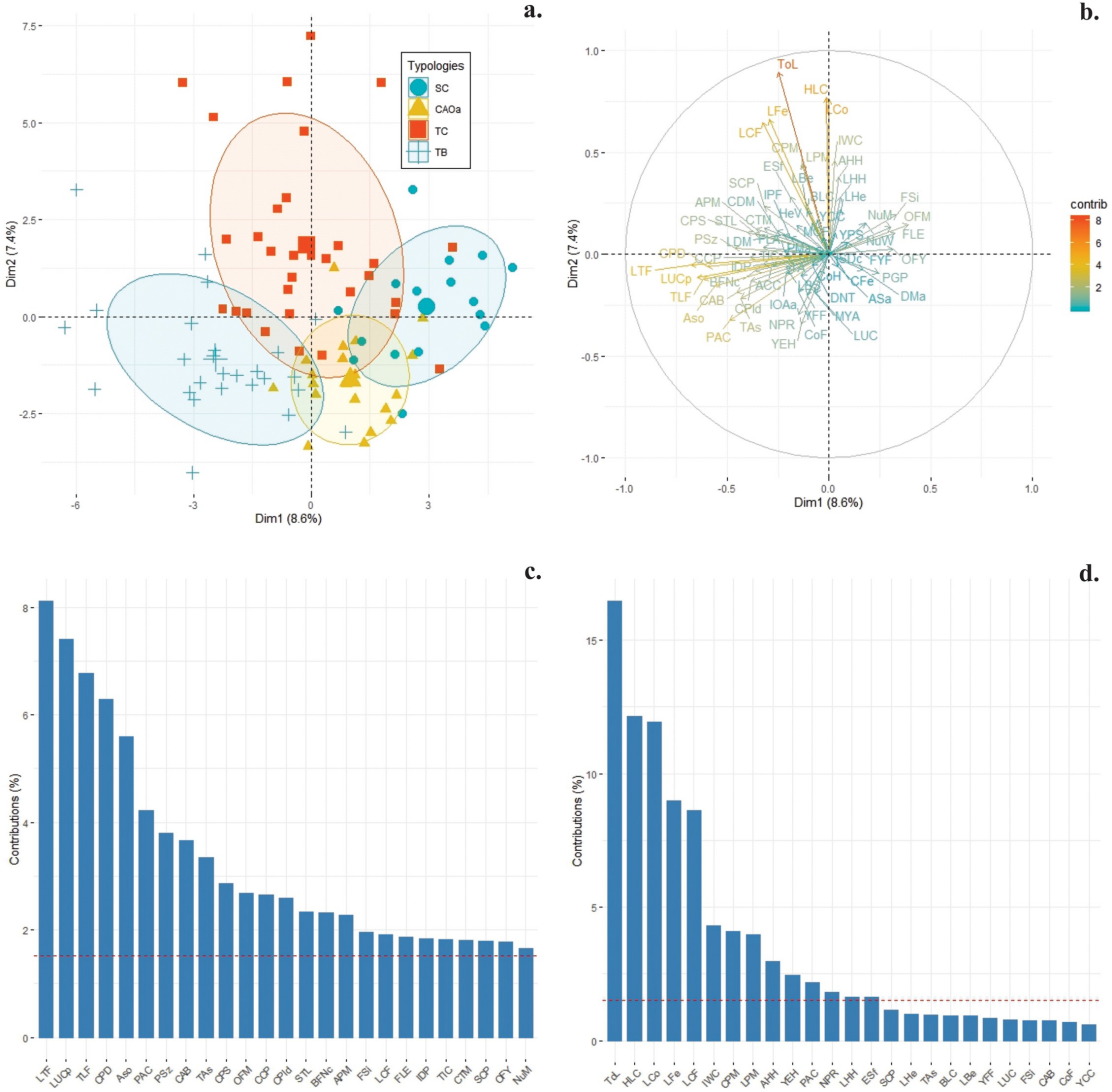
Typologies of coffee producers in southern Colombia. a. Small Conventional (SC), Conventional Associated with Organic approach (CAOa), Technified Conventional (TC), and Technified Business (TB). b. Contribution of each variable obtained through the analysis of principal components, c, and d: Variables with the greater contribution in principal component (PC) 1 and 2.

Based on a matrix of 66 variables characterized in each coffee producing household (Table 2) for each of the variables by capital and using cluster analysis, four typologies of coffee producers were identified (Table 3, Fig 1a): Small conventional (SC), Conventional Associated with Organic approach (CAOa), Technified conventional (TC) and Technified business (TB). Of the 66 variables, only nine allowed to differentiate the diverse typologies. For instance, only total labor force (ToL) and labor of fertilization (LFe) were different for CAOa with the other typologies for human capital. In the case of cultural capital, the type of organic fertilizers management (OFM) and organic fertilization dose per year (OFY) variables as technical assistance (TAs) of social capital allowed separating TB from the other typologies. In the case of political capital, the participation in activities of the community action board of the village (PAC) variable allowed the separation of SC from the different typologies, just as the distance nearest town (DNT) variable of physical capital allowed the separation of TC from the other typologies. Finally, both the certification of the coffee plantation (CCP) and labor cost of fertilization application (LCF) variables of financial capital allowed us to differentiate the CAOa typology from the others (Table 3). These typologies present higher and lower contributions (Fig 1b) in principal component 1 (Fig 1c) and principal component 2 (Fig 1d). The CAOa and TC types have medium capital endowments but were separated by Cultural capital variables, which are related to organic agriculture practices. According to the Monte-Carlo test, significant differences were found between the typologies of coffee producers (46% variance explained, p<0.001).

**Table 3:** The 66 variables characterized in each coffee producing farmers studied and their mean values in each type of the typology built. Values represent the mean ± standard deviation. The four types are: Small Conventional (SC), Conventional Associated with Organic approach (CAOa), Technified Conventional (TC), and Technified Business (TB). (LSD Fisher test significant at p <0.05).

Capital	variable	abbreviation	unity	SMALL CONVENTIONAL	CONVENTIONAL ASSOCIATED WITH ORGANIC APPROACH	TECHNIFIED CONVENTIONAL	TECHNIFIED BUSINESS	General	
				Mean S.D.	Mean S.D.	Mean S.D.	Mean S.D.	Mean S.D.	P-Valor
**HUMAN**	Age of head of household	AHH	Number of years	4.75 ± 3.31	42.65 ± 2.19	51.77 ± 2.48	45.85 ± 2.78	47.12 ± 1.38	0.1025
	Years of education of the head of household	YEH	Number of years	5.75 ± 1.17	6.08 ± 0.85	4.11 ± 0.47	6.90 ± 0.94	5.60 ± 0.42	0.1011
	Training Assistance	TA	Number of training courses	1.25 ± 0.40^b^	2.50 ± 0.25^a^	1.68 ± 0.23^b^	1.81 ± 0.27^a^^b^	1.82 ± 0.14	**0.032**
	Total labor force	ToL	Number of daily wages	22.91 ± 3.22^b^	14.35 ± 2.71^c^	44.33 ± 4.20^a^	22.96 ± 2.70^b^	28.22 ± 2.15	**<0,0001**
	Labor fertilization/year	LFe	Number of daily wages	13.59 ± 3.00^b^^c^	6.90 ± 1.64^c^	24.37 ± 2.94^a^	13.92 ± 2.01^b^	15.84 ± 1.45	**<0,0001**
	Labor application herbicide/year	LHe	Number of daily wages	1.25 ± 0.23	0.65 ± 0.15	1.13 ± 0.16	0.81 ± 0.15	0.96 ± 0.09	0.0901
	Labor disease management	LDM	Number of daily wages	0.94 ± 0.21^b^	1.10 ± 0.27^b^	1.97 ±0.29^a^	1.92 ± 0.24^a^	1.59 ± 0.14	**0.0148**
	Labor Pest Management	LPM	Number of daily wages	1.00 ± 0.39	1.50 ± 0.80	3.00 ± 1.20	1.73 ± 0.39	1.98 ± 0.46	0.5191
	Labor collection	LCo	Number of daily wages	5.06 ± 1.45^b^	3.40 ± 0.99^b^	12.00 ± 2.05^a^	3.65 ± 1.22^b^	6.62 ± 0.91	**0.0011**
	Labor Benefit	LBe	Number of daily wages	1.06 ± 0.11	0.80 ± 0.17	1.85 ± 0.49	0.92 ± 0.12	1.23 ± 0.18	0.0948
	Family Size	FSi	Number of persons	4.38 ± 0.39^a^	3.45 ± 0.31^a^^b^	3.58 ± 0.31^a^^b^	2.96 ± 0.19^b^	3.52 ± 0.15	**0.0398**
	Number of men	NuM	Number of men	2.69 ± 0.31^a^	1.50 ± 0.22^b^	1.87 ± 0.17^b^	1.65 ± 0.17^b^	1.87 ± 0.11	**0.0139**
	Number of women at home	NuW	Number of women	1.69 ± 0.22	1.95 ± 0.22	1.71 ± 0.22	1.31 ± 0.11	1.65 ± 0.10	0.1379
	Number of people who can read	NPR	Number of persons	4.19 ± 0.36^a^	3.30 ± 0.27^a^^b^	2.90 ± 0.28^b^	2.85 ± 0.21^b^	3.19 ± 0.15	**0.0211**
	Family’s average level of education	FLE	Number of years	6.14 ± 0.44^a^^b^	7.10 ± 0.71^a^	5.01 ± 0.40^b^	7.22 ± 0.55^a^	6.27 ± 0.28	**0.0056**
**CULTURAL**	Coverage Type Management	CTM	Number of coverages	0.56 ± 0.16	0.75 ± 0.14	0.71 ± 0.12	1.00 ± 0.18	0.77 ± 0.08	0.4089
	Soil conservation practice management	SCP	Number of practices	1.31 ± 0.25^a^	0.45 ± 0.15^b^	1.87 ± 0.29^a^	1.81 ± 0.33^a^	1.45 ± 0.15	**0.0017**
	Agroforestry practice management	APM	Number of practices	0.94 ± 0.23	1.30 ± 0.24	1.58 ± 0.20	1.85 ± 0.27	1.48 ± 0.12	0.1175
	Frequency per year of weed control chemically	YCC	Number of practices	1.06 ± 0.14	0.90 ± 0.19	0.87 ± 0.15	0.62 ± 0.18	0.84 ± 0.09	0.1134
	Frequency per year of fertilization of coffee plantations	FYF	Number of doses	3.25 ± 0.21	3.00 ± 0.21	3.26 ± 0.12	3.15 ± 0.12	3.17 ± 0.08	0.6138
	Availability of soil analysis	Asa	Number of analysis	0.56 ± 0.13	0.60 ± 0.11	0.32 ± 0.09	0.46 ± 0.10	0.46 ± 0.05	0.2082
	Frequency per year of foliar fertilization	YFF	Number of doses	0.38 ± 0.18^a^	0.85 ± 0.18^a^	0.39 ± 0.13^a^	0.88 ± 0.18^a^	0.62 ± 0.09	**0.0286**
	Type of organic fertilizers management	OFM	Number of types	0.88 ± 0.22^a^^b^	1.10 ± 0.18^a^	0.65 ± 0.14^b^	0.23 ± 0.08^c^	0.67 ± 0.08	**0.0003**
	Organic fertilization dose per year	OFY	Number of doses	0.75 ± 0.23^b^	1.70 ± 0.25^a^	0.81 ± 0.18^b^	0.08 ± 0.05^c^	0.78 ± 0.11	**<0,0001**
	Diseases management	DMa	Number of diseases	0.94 ± 0.06	1.00 ± -	0.94 ± 0.04	0.96 ± 0.04	0.96 ± 0.02	0.7137
	Pest Management	PMa	Number of pests	1.56 ± 0.27	0.90 ± 0.20	1.35 ± 0.21	1.62 ± 0.27	1.37 ± 0.12	0.2006
	Frequency per year of pruning to the shade	YPS	Number of pruning	0.81 ± 0.26	1.00 ± 0.19	0.71 ± 0.13	0.81 ± 0.15	0.82 ± 0.09	0.6348
	Pruning management to coffee plantation	MCP	Number of pruning	0.44 ± 0.13	0.65 ± 0.11	0.55 ± 0.09	0.46 ± 0.10	0.53 ± 0.05	0.5371
**SOCIAL**	Technical assistance	Tas	Number of attendances	0.38 ± 0.15^c^	1.05 ± 0.15^a^^b^	0.68 ± 0.10^b^^c^	1.27 ± 0.15^a^	0.87 ± 0.08	**0.0001**
	Benefits of the National Federation of Coffee Growers	BFNc	Number of benefits	0.94 ± 0.31^b^	1.80 ± 0.17^a^	1.90 ± 0.18^a^	1.85 ± 0.21^a^	1.70 ± 0.11	**0.0369**
	Associativity	Aso	Number of associations	0.56 ± 0.18^b^	1.60 ± 0.15^a^	1.42 ± 0.11^a^	1.77 ± 0.16^a^	1.41 ± 0.08	**<0,0001**
	Membership time in years to associations	MYA	Number of years	0.69 ± 0.25^b^	1.85 ± 0.18^a^	1.87 ± 0.17^a^	1.65 ± 0.17^a^	1.60 ± 0.10	**0.0004**
**POLITICAL**	Belonging to a community action board	CAB	Number of affiliations	0.44 ± 0.13^b^	1.00 ± -^a^	0.90 ± 0.05^a^	1.00 ± -^a^	0.87 ± 0.03	**<0,0001**
	Participation in activities of the community action board of your village	PAC	Number of units	0.38 ± 0.13^c^	0.95 ± 0.05^a^	0.65 ± 0.09^b^	0.96 ± 0.04^a^	0.75 ± 0.04	**<0,0001**
	Coffe producer ID	CPId	Number of IDs	0.44 ± 0.13^b^	1.00 ±-^a^	0.97 ± 0.03^a^	0.92 ± 0.05^a^	0.87 ± 0.03	**<0,0001**
**PHYSICAL**	Property Size	PSz	ha	4.68 ± 0.87^b^	5.26 ± 0.83^b^	5.87 ± 0.56^b^	9.95 ± 1.20^a^	6.68 ± 0.49	**0.0025**
	Distance nearest town	DNT	km	8.75 ± 1.71^b^^c^	12.50 ± 1.37^a^	7.31 ± 0.74^c^	11.00 ± 1.28^a^^b^	9.70 ± 0.63	**0.0111**
	Level of basic services	LBS	Number of services	2.94 ± 0.06	3.00 ± 0.07	2.87 ± 0.08	3.12 ± 0.13	2.98 ± 0.05	0.3858
	The technological level of the farm	TLF	Number of technologies	3.75 ± 0.38^b^	3.45 ± 0.39^b^	4.13 ± 0.38^b^	7.62 ± 0.51^a^	4.89 ± 0.28	**<0,0001**
	Level of tools on the farm	LTF	Number of tools	22.56 ± 1.84^b^	23.50 ± 1.78^b^	26.55 ± 1.76^b^	48.08 ± 4.37^a^	31.23 ± 1.80	**<0,0001**
	Structural technological level	STL	Number of technologies	3.81 ± 0.61	4.10 ± 0.36	4.68 ± 0.33	4.50 ± 0.32	4.35 ± 0.19	0.3544
**NATURAL**	Process that generates pollution	PGP	Number of processes	0.88 ± 0.24^a^	0.90 ± 0.19^a^	0.39 ± 0.12^b^	0.38 ± 0.14^b^	0.58 ± 0.08	**0.0142**
	Ecosystem services provided by the farm	ESf	Number of services	3.00 ± 0.48	2.15 ± 0.39	2.74 ± 0.32	3.15 ± 0.39	2.77 ± 0.19	0.2704
	Land use for crops	LUC	Ha	0.75 ± 0.22	1.71 ± 0.51	1.20 ± 0.19	0.98 ± 0.17	1.17 ± 0.14	0.3763
	Forest land use	FLA	Ha	0.69 ± 0.38^b^	0.46 ± 0.12^b^	1.01 ± 0.34^b^	2.51 ± 0.74^a^	1.25 ± 0.26	**0.0073**
	Land use for coffee production	LUCp	Ha	2.37 ± 0.28^b^	3.22 ± 0.43^b^	3.57 ± 0.42^b^	7.32 ± 0.85^a^	4.34 ± 0.35	**<0,0001**
**FINANCIAL**	Hectare value	HeV	thousands of dollars	20.06 ± 2.43	18.86 ± 1.58	24.68 ± 1.75	21.01 ± 2.20	21.61 ± 1.02	0.1153
	Cost Property size	CPS	thousands of dollars	98.16 ± 25.29^b^	90.12 ± 15.08^b^	151.18 ± 19.59^a^	204.10 ± 33.54^a^	143.72 ± 13.31	**0.0067**
	Coffee production	CPD	Ha	1.91 ± 0.31^b^	2.61 ± 0.47^b^	2.70 ± 0.38^b^	4.88 ± 0.69^a^	3.15 ± 0.28	**0.0027**
	Access to credits	ACC	No. of credits	0.44 ± 0.13^b^	1.10 ± 0.10^a^	0.94 ± 0.14^a^	1.04 ± 0.17^a^	0.91 ± 0.08	**0.007**
	Certification of the coffee plantation	CCP	No. of certifications	0.31 ± 0.18^b^^c^	0.00 ± 0.00^c^	0.52 ± 0.17^b^	1.19 ± 0.28^a^	0.56 ± 0.11	**0.0001**
	Labor cost of fertilization application	LCF	thousands of dollars	0.15 ± 0.03^b^^c^	0.08 ± 0.02^c^	0.30 ± 0.04^a^	0.17 ± 0.03^b^	0.19 ± 0.02	**<0,0001**
	Cost of labor handling herbicides	LHH	thousands of dollars	0.01 ± 0.00	0.01 ± 0.00	0.01 ± 0.00	0.01 ± 0.00	0.01 ± 0.00	0.0864
	Labor cost diseases management	CDM	thousands of dollars	0.01 ± 0.00^b^	0.01 ± 0.00^b^	0.02 ± 0.00^a^	0.02 ± 0.00^a^	0.02 ± 0.00	**0.0068**
	Cost of labor pest management	CPM	thousands of dollars	0.01 ± 0.00	0.02 ± 0.01	0.04 ± 0.02	0.02 ± 0.00	0.02 ± 0.01	0.4655
	Harvest labor cost	HLC	thousands of dollars	0.06 ±0.02^a^^b^	0.04 ±0.01^b^	0.14 ± 0.02^a^	0.04 ± 0.01^b^	0.08 ± 0.01	**0.0013**
	Benefit labor cost	BLC	thousands of dollars	0.01 ± 0.00	0.01 ± 0.00	0.02 ± 0.01	0.01 ± 0.00	0.01 ± 0.00	0.0852
	Income for wet coffee/farm	IWC	thousands of dollars	0.96 ± 0.67	0.76 ± 0.51	4.99 ± 1.73	0.73 ± 0.38	2.19 ± 0.63	0.1505
	Income for dry parchment coffee/farm	IDP	thousands of dollars	7.26 ± 2.14	8.86 ± 3.03	17.90 ± 3.93	32.13 ± 10.88	18.10 ± 3.50	0.2071
	Cost of fertilizers	CFe	thousands of dollars	1.05 ± 0.11	1.15 ± 0.05	1.28 ±0.05	1.09 ± 0.09	1.16 ± 0.04	0.485
	Income from other agricultural activities	IOAa	thousands of dollars	0.04 ± 0.02	0.08 ± 0.06	0.06 ± 0.03	0.35 ± 0.21	0.14 ± 0.06	0.8371
	Income for special dry parchment coffee/farm	ISDc	thousands of dollars	- ± -	0.05 ± 0.05	0.48 ± 0.37	- ± -	0.17 ± 0.13	0.2566
	Income for pasilla/farm	IPF	thousands of dollars	0.09 ± 0.04	0.11 ± 0.06	0.19 ± 0.07	0.14 ± 0.09	0.14 ± 0.04	0.648
	Total income for coffee	TIC	thousands of dollars	8.34 ± 2.36^b^	9.86 ±3.02^b^	23.62 ± 4.25^a^^b^	33.34 ± 10.78^a^	20.75 ± 3.53	**0.0192**
	Cost of herbicides	CoH	thousands of dollars	0.00 ± 0.00	0.01 ± 0.00	0.01 ± 0.00	0.01 ± 0.00	0.01 ± 0.00	0.2922
	Costs of fungicide	CoF	thousands of dollars	0.02 ± 0.01	0.02 ±0.01	0.02 ± 0.01	0.02 ± 0.01	0.02 ± 0.00	0.7803

^a, b, c:^ Averages with a letter in common between rows are not significantly different at 5% probability.

#### Small Conventional (SC) (17% of families)

This family is specifically differentiated from the other typologies by having a higher average number of people per family (FSi) and the highest number of people per family who can read (NPR). Likewise, they are households that have a greater number of women in their family nucleus (NuW), a variable part of the human capital. However, this typology presents the least frequency in technical assistance (TAs social capital) and little participation in the communal action board the village (PAC) that is part of the political capital. The economic income of these families does not depend exclusively on the coffee cultivation, therefore, this crop is not its main livelihood. These families need to sell their labor force to generate economic income to supply other basic needs. This is also due to the small area of land available to diversify production, a situation that has made it impossible to access bank loans and the possibility of being able to certify coffee plantations. These are families that present a low technological level with the essential elements for the development of the coffee activity. They use few techniques in the conservation of the soil and the management of the residues resulting from the coffee activity.

#### Conventional associated with organic approach (CAOa) (22% of the families)

The main characteristics that differentiate it from the other typologies correspond to handling organic practices to coffee cultivation. This is mainly related to the fertilization of the coffee plants with organic fertilizers (OFM) with a greater frequency than the other typologies (OFY). At the level of each capital, for example, in the *Human* capital, the availability of labor is meager; however, the level of education is secondary with a higher amount of assistance to training that is translated at the level of the *Cultural* capital in practices of organic management, which is the main characteristic of this typology. At *Social* and *Political* capital, a significant presence in technical assistance processes was found. Besides, recognition of the associativity in terms of experience (associated time), membership and participation in processes carried out by the community action board, as well as the possession of a coffee card that identifies the association as part of the National Federation of Coffee Growers (FNC) was also higher. Being part of the coffee guild (FNC) allows them to access benefits from the government channeled by this organization. As for physical capital, it is a typology with a low technological level, having the basic elements for developing the coffee activity, using few techniques in the conservation of the soil, and small areas of forest. Regarding financial capital, this type has had access to more than one credit with a financial entity; however, it does not have any certification about the productive activity. It also has the lowest labor costs for fertilizer application, disease management, and grain collection.

#### Technified conventional (TC) (33% of the families)

This typology of coffee families is characterized by having the highest investments in labor costs related to the harvesting of coffee beans as well as in the fertilization of the crop. All these activities have an impact on financial capital, increasing costs. These are families with a basic primary education level and are the ones closest to the population center. At the level of *Cultural* capital, they present the highest amount of soil conservation practices; however, the amount of coverage in the coffee plantation is low compared to the other typologies. It corresponds to the typology that has the most years of association with the greatest amount of benefits from the FNC and belongs to and participates in the community action board’s activities. Regarding *Physical* capital, it is a type with a medium level of technology, which means that it possesses some additional elements that have allowed it to technify coffee activity. In the *Natural* capital, this typology has the lowest pollutant load because it has processes related to the adequate handling of the garbage, septic tank, and coffee honey management.

#### Technified business (TB) (28% of the families)

These are families whose average education is higher than the other typologies, focusing this education mainly on the management of crop diseases. The average number of people who make up this typology is low compared to the others, which is because some of them live in the population centers. At the level of the Cultural capital, it has a greater amount of soil conservation practices, and by their economic capacity, they develop practices of constant fertilization to the crop; nevertheless, they do not handle organic agriculture processes. Its Financial capital is high compared to the other types. Hence, its farm works as a company, backed by high income (from the sale of dry parchment coffee and pasilla coffee) that has allowed economic solvency to access credit, thus being able to certify their coffee plantations. The producer’s financial capacity into this typology of farm has allowed them to get the highest Physical capital in terms of the level of technology and tools availability. Its Social and Political capital has allowed it to receive many benefits from the National Federation of Coffee Growers and the community action board. Its high level of production combined with the Political capital has allowed it to have access to more frequent technical assistance that translates into the implementation of practices to manage diseases.

### Synergies and trade-offs between livelihood capitals

**Fig 2:**
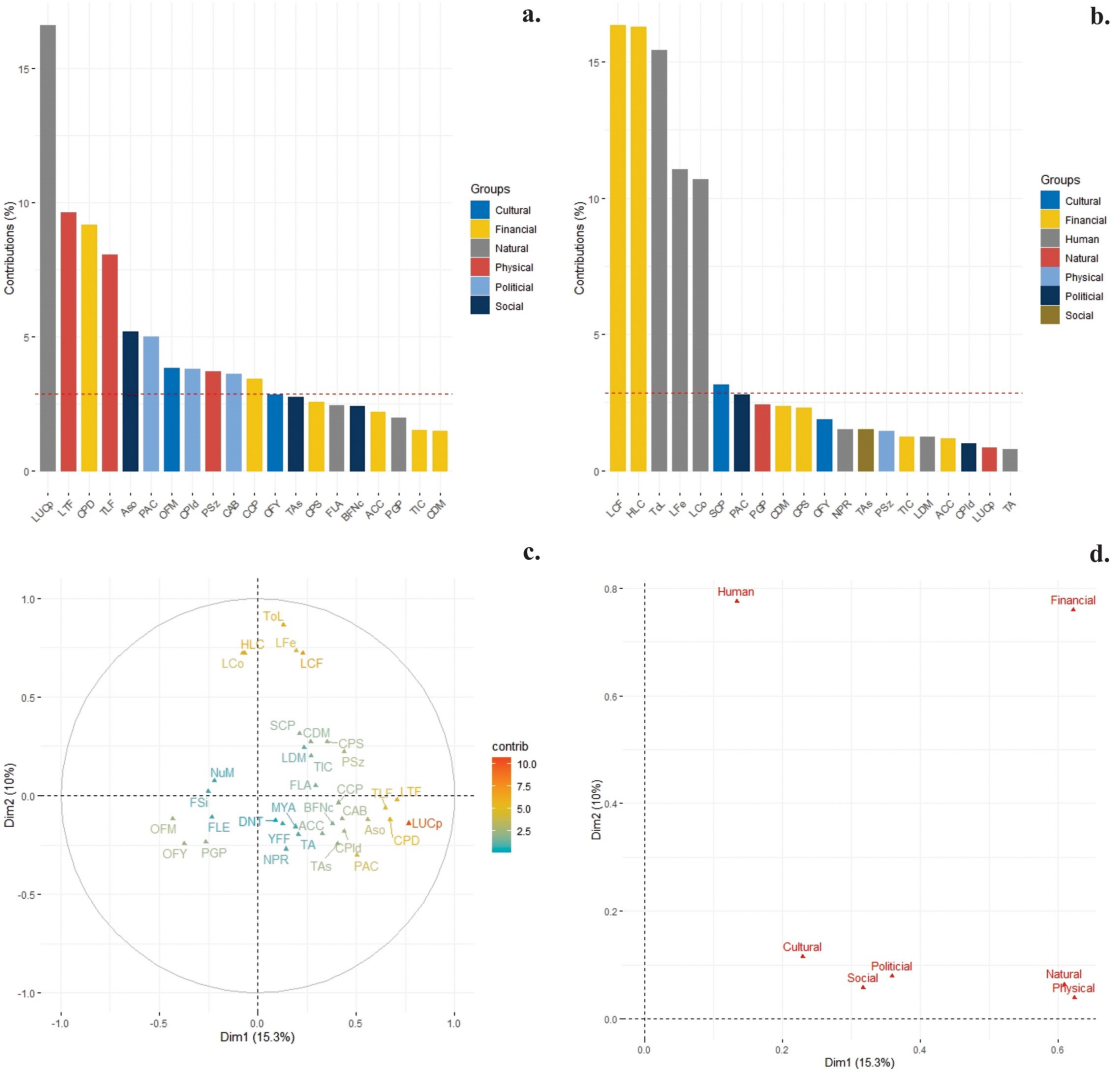
Most important variables for each capital that impact the Community Capital Index (CCI). a and b: Contribution of the variables for each capital in principal component (PC) 1 and 2. The variables above the dotted line presented the greatest contribution, c: distribution of variables on a biplot plane obtained through a principal component analysis, d: synergies and trade-offs between capitals of the communities obtained through multiple factor analysis.

From the total of variables used (66) for the generation of coffee producers’ typologies, 35 presented significant differences between the typologies. From them, only organic fertilizers management (OFM), organic fertilization dose per year (OFY), technical assistance (TAs), participation in activities of the community action board of the village (PAC), distance to nearest town (DNT), certification of the coffee plantation (CCP) and labor cost of fertilization application (LCF) were the most relevant variables that allowed generating the differences between the typologies. In this sense and taking as a reference the total of variables used for the characterization, 100% of variables of the *Social* and *Political* capital presented significant differences between the typologies, being these the capitals most responsible for the differences between coffee families (Fig 2a and 2b).

Likewise, the *Cultural*, *Financial*, and *Human* capital presented 60, 42, and 28% of its variables with significant differences, respectively. Of these 35 variables and according to the results obtained by the multiple factor analysis, there was a greater contribution of 12 and 7 variables in component one and two, respectively, which were mostly Political capital (4 variables), Financial capital (4 variables), Social capital and Human capital (3 variables). Among capitals, those that presented the highest quality of representation on the factorial map in components one and two were Physical and Human, respectively. The variables that showed greater incidence on the relationship between capitals were the area in use for coffee cultivation (LUCp), followed by the level of tools on the farm (LTF) and the production of coffee (CPD) that represent Natural capital, Physical capital and Financial capital, respectively. Likewise, in component two, variables from *Financial* capital (i) cost of labor for fertilization (LCF) and (ii) harvesting (HLC), and from *Human* capital (i) total labor (ToL), the (ii) amount of wages for fertilization (LFe) and (iii) harvesting of grains (LCo) presented a greater incidence. The variables mentioned above have allowed capitals such as *Social* and *Political* to have synergies, whose function is to increase the well-being of families based on the action or capacity of two or more capitals.

Likewise, for coffee families to increase in *Financial* capital, they must have synergies simultaneously with *Social* and *Political* capital. At the level of trade-offs between capitals, no statistical evidence was found that would demonstrate that increasing one capital would negatively affect another. Therefore, it is evident that to move from a typology such as "small conventional" to another with better well-being, all capitals should be geared together, thus improving processes (variables) that generate positive effects on the Community Capital Index (CCI), in agreement with the essence of the spiral theory. According to the co-inherence analysis, a greater significant covariance was found for *Financial* capital with *Human*, *Physical*, *Natural*, *Social*, and *Political* capital (Equity >0.09, value p = 0.001, Monte de Carlo test). Among the highest covariances found among capitals, the one presented in *Social* and *Political* capital stands out (RV = 0.35, value p = 0.001, Monte de Carlo test).

**Fig 3:**
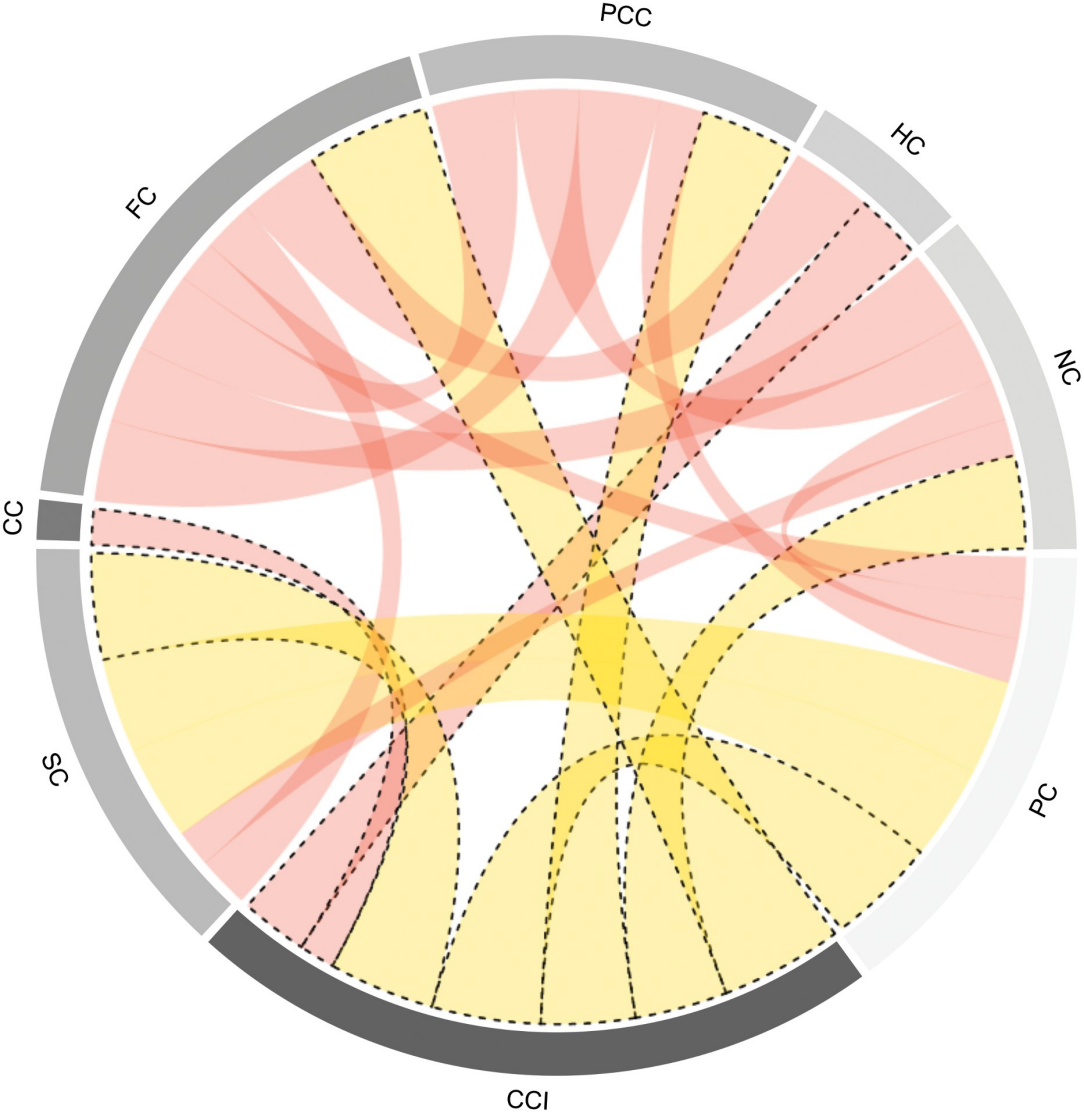
Pearson’s correlation coefficients between the Community Capital Index (CCI) and each capital. All ribbons within the circle correspond to significant correlations with Pearson coefficients greater and less than 0.5 in yellow and red, respectively. The width of the ribbon means the degree of relationship, the closer to one is wider. CC: *Cultural* Capital, FC: *Financial* Capital, PCC: *Physical-Constructed* Capital, HC: *Human* Capital, NC: *Natural* Capital, PC: *Political* Capital, SC: *Social* Capital.

In this respect, some capitals showed a higher ratio of CCI (yellow ribbons), such as PC (*Political* Capital) and SC (*Social* Capital), among others (Fig 3). However, as capitals significantly correlated with CCI, this was not as high (red ribbons). This analysis allowed us to confirm the strength of the *Social* and *Political* capitals on the CCI, previously found in the multiple factor analysis (Fig 1d).

### The well-being of coffee-growing families: Community Capital Index and capital spiral

**Fig 4:**
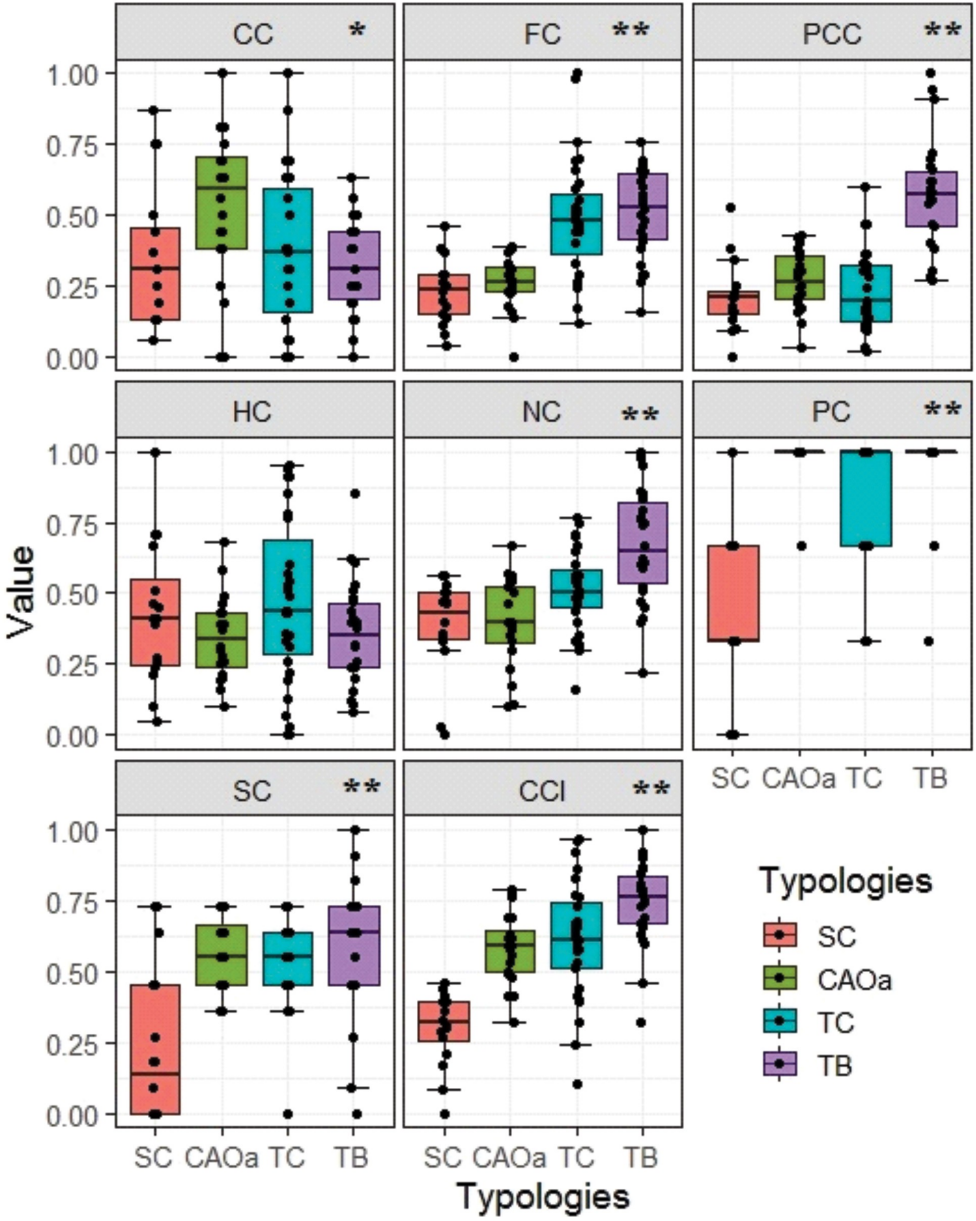
Value of the indicator for each capital. (CC: *Cultural* Capital, FC: *Financial* Capital, PCC: *Physical-Constructed* Capital, HC: *Human* Capital, NC: *Natural* Capital, PC: *Political* Capital, SC: *Social* Capital) of the community and for the Community Capital Index (CCI). Small conventional (SC), Conventional associated with organic approach (CAOa), Technified conventional (TC), and Technified business (TB).

When analyzing the sub-indicators created for each capital, significant differences were found for *Cultural*, *Financial*, *Physical-Constructed*, *Natural*, *Political*, and *Social* capitals among the typologies. No significant differences were found for *Human* capital (Fig 4). Following the order of technological and business development in the typologies of coffee producers, it was found that the *Financial*, *Physical*, *Natural*, *Political*, and *Social* capital increase their value from the Small Conventional (SC) typology to the Technified Business (TB), which translated into a higher value in CCI. At the level of *Cultural* capital, the CAOa typology presented the highest value being different from the other typologies (p<0.05). The index’s value went from 0.30 to 0.73 for SC to TB, respectively (Fig 4).

To improve the CCI value, it was necessary to make specific changes in some variables that make up the capitals. As a result of these changes, 32 variables were found to have a positive (21 variables) and negative (11 variables) impact. Four of the 32 variables were classified as "generalist" due to the high incidence of family mobilization among the typologies both upwards and downwards, including (i) the availability of labor (*Human* capital), the dose of organic fertilization per year (*Cultural* capital), (iii) participation in activities of the community action board of your village (*Political* capital), and (iv) the distance from the nearest population center (*Physical* capital).

When analyzed the mobility of coffee growing families between typologies, we found that the basis for the mobilization of the Small Conventional (SC) to Conventional associated with organic approach (CAOa) were the *Social* capital (Association), the *Political* capital (Participation in the activities of the community action board, Coffee producer ID), and the *Financial* capital (access to bank credits). All of the variables which presented a positive effect, demonstrating the simultaneous synergy of *Social* and *Political* capital with *Financial* capital. However, the absence of women in the household, labor availability (*Human* capital), and the lack of management practices in soil conservation (*Cultural* capital) do not allow this change in typology.

Following the upward moving in the spiral based on the CCI to move from CAOa to TC, it is required a high investment in *Financial* capital. This investment must be made by performing certification processes of their coffee plantation, increasing labor for the application of inputs (fertilizers), managing diseases, and harvesting grains. All those actions are translated into rising coffee production and, therefore, in the income for its sale. The promotion of CAOa to TC requires soil conservation practices, which is a process in the *Cultural* Capital. However, this transition from CAOa to TC may be affected if the TC typology does not improve processes to reduce contamination (*Natural* capital) and procedures related to organic crop management (OFY and OFM variables of the *Cultural* capital). Finally, in the ascent of a coffee family from TC to TB, there is evidence of the technological increase (TLF and LFT in the *Physical* capital) in the development of the activities that affect coffee production. It is observed that a variable that affects the increase in the Community Capital Index (CCI) is the possession of forest areas (FLA of *Natural* capital), an important characteristic in the certification processes (CCP of *Financial* capital). We found a high relationship between the level of education in the family (FLE for human capital), the importance of training (TA), and technical assistance on the farm (FAT) as a knowledge base, with the possible increase in well-being. However, when the technification processes were presented, the inefficiency in the application of fertilizers (LFe) and harvesting processes (LCo) exhibited a negative impact on coffee families that are interested in participating in business processes (TB). Similarly, adverse effects were observed by the lack of organic management schemes for the crop (OFY and OFM variables of the *Cultural* capital).

## Discussion

### Typologies of coffee producers in southern Colombia

We identified four different typologies with specific characteristics of coffee producers from the south of Colombia: Small conventional (SC), Conventional associated with organic approach (CAOa), Technified Conventional (TC), and Technified Business (TB). Technological differences between the typologies were found, which affected the agronomic yield of coffee cultivation and its profitability. Similar to our findings, different studies [8,9,34–36] have identified that variables such as educational level, extension services, access to credit, consolidation of land tenure, improved variety of coffee trees, cultivation system, relationship at the level of community action boards, and the level of associativity, significantly improve the level of well-being of coffee families. In particular, in our study, the main characteristics that contribute to the improvement of life quality are linked to extension services and governance mechanisms that have allowed the empowerment of the coffee activity. This result is consistent with Ngango and Kim [34], who reported that extension is a fundamental part of achieving intensification of production. Likewise, Hajjar et al. [37] and Elder [38] have found that the increase in sustainability is due to the transferability of governance mechanisms for decision making. One explanation for this finding is that the consolidation of governance from the community action board has allowed the channeling of subsidies to fertilizers, credit facilities, access to marketing, and rural infrastructure development. Therefore, coffee families have been able to take advantage of such schemes to improve their efficiency and productivity, a situation that has been described by Zeweld et al. [39], who mentions that there is an impact of agricultural practices on crop yields and farmers’ means. It is also highlighted that education and training are determinants of food security’s positive results at the household level as part of the social capital, a situation previously described by Sseguya et al. [40].

In coffee production, there are typical practices related to intensification, such as reducing shade levels by eliminating the accompanying trees [41,42], a practice that was not found on our farms since most are in the process of certification with the Rainforest Alliance, UTZ, and others. Instead, we found typologies of small coffee producers that have implemented agroecological practices in their agroforestry systems, such as reducing dependence on agrochemical inputs by implementing organic processes and increasing the diversity of shade trees that are the result of certification schemes and translates into income diversification. These decisions are related to maximizing productivity from the natural assets on the farm that has allowed to promote more environmentally friendly agricultural practices such as the adoption of organic practices. This is due to the membership of farmers’ cooperatives that encourages the adoption of certification. As in most of the world, coffee growing families in southern Colombia have found that the well-being and sustainability of households are related to the diversification of livelihoods to obtain additional sources of income [35,43–45]. In this sense, whether families have an alternative source of income through livelihood diversification is very important for the sustainability of household livelihoods, especially for low-income farming groups, smallholders, and marginal farmers, such as shifting cultivators, who live on disadvantaged land with poor infrastructure and limited connectivity [46].

### Synergies and trade-offs between livelihood capitals

As mentioned in different studies, *Human* capital is considered an asset that influences livelihoods [17,25,27]. Interestingly, we found that in addition to *Human* capital (total labor, labor for fertilization, and labor for coffee harvesting), *Social* capital (association) and *Political* capital (participation in the activities of the community action board of the village) were the basis for development. *Social* capital and *Political* capital also allowed the typification of coffee-growing families. These capitals were the basis for the technological and productive development and the increase in the well-being of coffee growing families in the south of Colombia, simultaneously influencing the generation of synergy with *Financial* capital, which, in turn, influences the Community Capital Index. Usually, The literature mentions that the high use of technologies for coffee production, such as production in full sun with high demand for fertilizers, has led to a decrease in the biodiversity of accompanying trees, which provide some regulation and cultural services [47,48]; however, the implementation of this practice has not been found in our typologies of coffee producers. Nevertheless, we observed that the use of organic management technologies resulting from the implementation of certifications to achieve environmental and economic benefit [49–51] demonstrates the relationship for those farmers who attend training in soil conservation methods [52].

Furthermore, we found that *Social* capital contributes to household resilience due to the generation of associative processes, in addition to the synergy of *Political* capital. In this sense, *Social* capital by itself is insufficient and must be accompanied by *Political* capital, which complements what is described by Sseguya et al. [40]. It has also been mentioned that *Social* capital has positive impacts on the adoption of agricultural practices on farms [39]. In the case of the coffee families studied, this capital has contributed to the resilience of the households, facilitating the diversification of livelihoods through access to resources and technical assistance services and access to benefits through the coffee cedula of the National Federation of Coffee Growers. The above shows the relationship between state institutions and communities with the common objective of managing resources to respond to emergencies, both economic and natural, a situation that has been described by Baumman and Sinha [53]. However, in the absence of any state support for the development of the coffee activity, this can be considered a driver of rural abandonment [54]. The growth effect of *Social* capital is manifested in how networks and trust facilitate access to productive resources and the exchange o knowledge [12] among coffee families. For the above to exist, *Political* capital must be distributed in a legitimate manner using social power (political positions such as in the community action board) to spread the different means equitably without subordination between the capitals as reported by Mbiba et al. [55].

In the coffee families analyzed in our study, the synergy between the capitals has contributed to a "healthy ecosystem", which has translated into a "vibrant economy" with "healthy and happy people", which has allowed increased well-being. Therefore, we show clearly how the existence and flow of social and political capital can drastically alter the success of the coffee community, being these the main upward spiral drivers. Social and Political capital were the vital capitals for the success of the community; the lack of social capital can make the community collapse, while high social capital can make it grow. This is supported by Flora [56] and Emery and Flora [25], who indicate that the effect of the spiral is based on the synergy between the capitals, which is complex and depends mostly on the context of the community.

### The well-being of coffee-growing families: Spiral of capitals and Community Capital Index (CCI)

With the application of the spiral of capitals, different variables were identified to increase and decrease the well-being of families measured by the Community Capitals Index (CCI) proposed in this study. The spiral approach allowed us to recognize the importance of the balance and synergies between assets or capitals in sustainable rural development processes [24,25]. Similarly, we were able to understand how the community organizes itself to generate development and entrepreneurship as a social and financial strategy [57]. Our results indicate that when the social capital is strengthened with the linking of associations and groupings, there is a higher possibility that the producer would continue and remain in their activity with a greater probability of success. Hence, it allowed families with low levels of well-being (SC) to climb to higher levels of technology and entrepreneurship (TB), which translates into a more significant capital endowment and greater well-being. According to our findings, for the increase in well-being is necessary the women’s participation in decision-making and the development of typical coffee-growing activities. On the other hand, based on DFID’s Sustainable Livelihoods Approach, the indicator has been constructed as a manner to measure well-being. The livelihood approach has also been used in other studies to determine the incentives, restrictive, and regulatory capitals that influence decisions [58]. It has also been used to measure different adaptation strategies, showing the positive impact of *Natural* and *Social* capital on farmers’ decisions on climate change adaptation strategies [59].

### Implications of coffee policies on the increase of well-being

Promotion of the strengthening of the capacities of the associative groups, community groups, and cooperatives of coffee growers has been part of the policy of the Colombian National Federation of Coffee Growers (FNC, its Spanish acronym), which has translated into the reduction of the basic unsatisfied needs. This leads to coffee-growing families find themselves with better living conditions than the non-coffee growers. Likewise, the efforts of the coffee institutions (community action boards, municipal coffee committees) have been directed towards strengthening not only the productive apparatus of Colombian coffee-growing, but also towards the sustainability of the sector and the improvement of the quality of life and well-being of thousands of rural inhabitants. Our study found that the basis of development and growth in well-being is the social and political capital, probably due to all the work and development of activities carried out by the extension service as part of the governance and capacity building policy of the FNC. Nevertheless, we demonstrated marked differences in the degree of technology and technological development that has translated into the efficiency of production, which affects profitability. Overall, our results show that the application of different coffee politics in the last years has positively impacted the well-being of the coffee families, considering the base of the coffee ecosystem to the social and political capital.

We found that social and political capitals have a positive effect on the decisions of coffee families in terms of life strategies. In this sense, it is recommended that coffee-growing families form and participate in producer associations and community action boards in their villages. This process will allow them to design strategies and join forces to be more competitive in the coffee industry. The benefits of the associativity will be translated into a better community organization that increases the integration between the families, increases and improves their productivity, reduces costs, and achieves real access to markets. In more specific terms, the association will be related to the business initiative that will allow the development of collaborative economic actions in order to receive incentives for differentiated products placed in the market. Likewise, community organizations can be important levers for transformational change since they can influence how power structures are shaped in the community. At the policy level, we can recommend that strengthening community organizations to be bridge actors and leveraging adequate support and resources from all levels and sectors for implementation can have transformational impacts on the lives of coffee farmers. These include fostering collaborative and flexible arrangements, linking people and organizations vertically and horizontally, building adaptive capacity to identify interventions that protect livelihoods and ecosystems in a changing climate. Certification of production under different labels will allow access to specialty coffee markets, allowing for changes at the process level and in the form that coffee is produced. During this change, producers will acquire skills that will allow them to direct their different assets and turn coffee production more sustainable. These certifications benefit the producers since they gain access to information, technology, social networks and resources that they did not have before obtaining the certification. For that reason, we recommend that at the moment of making interventions in the coffee landscape of any place worldwide, important processes such as producer associations, community boards, and certified/organic production are intervened.

## Conclusions

The Community Capital Index (CCI) proposed in our study allowed us to accurately measure well-being using the capitals framework within the livelihoods approach, finding that main capitals that allowed for differences in coffee families (i. Small Conventional SC, ii. Conventional associated with organic approach CAOa, iii. Technified Conventional TC and iv. Technified Business TB) were social and political. The well-being measured by the proposed CCI showed that the increase in sustainability is achieved from the production of certified coffee, with technification in the production and development of the activity in a business manner.

The associativity variable that starts with political capital allowed for an increase in the well-being of some coffee growing families within the framework of the spiral of community capitals. This asset allowed canalizing technical assistance, benefits as far as inputs and qualification that made possible synergies with the financial capital, increasing the knowledge in the coffee cultivation and, therefore, its production, situation presented in the typologies Small Conventional (SC) and Technified Business (TB). We also found that the technological level of coffee families is related to the level of poverty, not only financial scarcity (economic dimension/assets focus) but also poverty in the dimensions of capabilities and exclusion (especially Cultural and Political capital). All this leads to increased levels of uncertainty, calculated through the Community Capital Index (CCI). The above situation was specifically presented in the Small Conventional (SC) typology, where coffee cultivation is not its main livelihood and the producers need to sell their labor force to generate economic income to supply other basic needs. Consequently, the opportunity cost related to the *Financial* and *Social* capital does not allow them to achieve the development of capital as the *Political* one, which is crucial for the development of their livelihood.

In order to increase the CCI, it is necessary to make substantial changes at the community level, specifically in "generalist" variables that influence the ascending (upward) and descending (downward) mobility of coffee families between different typologies, being (i) the availability of labor (Human Capital), the dose of organic fertilization per year (Cultural Capital), (iii) the participation in activities of the community action board of their town (Political Capital), and (iv) the distance from the nearest population center (Physical Capital) the variables that allow to increase or reduce well-being.

## Supporting information

S1 File Variables of each of the community’s capitals. (XLSX)
